# Differentiation of ripe and unripe fruit flour using mineral composition data—Statistical assessment

**DOI:** 10.1016/j.dib.2020.105414

**Published:** 2020-03-12

**Authors:** Abbas F.M. Alkarkhi, Wasin A.A. Alqaraghuli, N.R. Mohamed Zam, D.M.A. Manan, M.N. Mahmud, Nurul Huda

**Affiliations:** a Universiti Kuala Lumpur Business School (UniKL Bis), 50250, Kuala Lumpur, Malaysia; b Skill Education Center, PA, A-07-03 Pearl Avenue, Sungai Chua, 43000 Kajang, Selangor, Malaysia; c Universiti Kuala Lumpur Branch Campus Malaysian Institute of Chemical & Bioengineering Technology (UniKL, MICET), 78000 Melaka, Malaysia; d Faculty of Food Science and Nutrition, Universiti Malaysia Sabah, 88400 Kota Kinabalu, Sabah, Malaysia

**Keywords:** Major element, Minor element, Heavy metal, Discriminant analysis, Cluster analysis

## Abstract

Data on the mineral composition and content of one heavy metal measured in three different fruit flours prepared from ripe and unripe fruits (pulp and peel) are presented. The mineral composition (sodium (Na), potassium (K), magnesium (Mg), calcium (Ca), zinc (Zn), copper (Cu), iron (Fe) and manganese (Mn)) and content of one heavy metal (lead (Pb)) of the flours were analyzed by atomic absorption spectrophotometry. The analysis showed that the data can be used for differentiation between different fruits and stages of ripeness, as revealed by discriminant analysis and cluster analysis. The data provided can be used by researchers and scientists in the differentiation of fruits based on major and minor mineral elements.

Specification table

**Table:** 

Subject area	*Food chemistry*
More specific subject area	*Major and minor mineral elements and a heavy metal*
Type of data	*Figures, tables*
How data was acquired	*- Atomic absorption spectroscopy (AAS)* *- Periodate oxidation method*
Data format	*Raw, analyzed*
Parameters for data collection	*The samples were washed, cleaned, peeled, cut, dried at* 60 °C, *ground, and sieved*
Description of data collection	*Samples of three types of fruits (mango, pineapple, and papaya) and different stages of ripeness (unripe and ripe) were collected. The samples were analyzed for mineral composition.*
Data source location	*Malaysian Institute of Chemical & Bioengineering Technology Universiti Kuala Lumpur, (UniKL, MICET)*
Data accessibility	*The raw data are provided as a supplementary data with this article.*
Related research article	*Trilícia et al.**[5]*, *Differential contribution of grape peel, pulp, and seed to bioaccessibility of micronutrients and major polyphenolic compounds of red and white grapes through simulated human digestion. Funct. Foods 52, 699–708*

## Value of the data

• The investigated data highlight the changes in major and minor mineral elements and one heavy metal in unripe and ripe fruits (three types of fruits), which is valuable for studies working on mineral and trace elements.
• The present data on major and minor elements and one heavy metal provide information for identification of the stage of ripeness and type of fruit, which can be ultimately used by researchers and scientists in the discrimination of fruits based on major and minor mineral elements.
• The investigated data highlight the relationship between different minerals to describe the behavior of these elements.

## 1 Data

The raw data in this article represent major and minor mineral elements and one heavy metal measured in three types of fruit (mango, papaya and pineapple). The raw data are provided as supplementary data, and the raw data are presented in Table 1 as the mean and standard deviation. Moreover, the data set includes the outputs of discriminant analysis, including the eigenvalues and proportion of variance explained by each discriminant function (Table 2). Furthermore, the outputs for the first three discriminant functions are presented in pictorial form. The data also include information on the similarities and dissimilarities as a result of cluster analysis, presented in pictorial form. The abbreviations for the data presented in tables and figures are as follows: RPUP: ripe pulp pineapple; RPEP: ripe peel pineapple, GPUP: unripe pulp pineapple, GPEP, unripe peel pineapple, RPUM: ripe pulp mango; RPEM: ripe peel mango, GPUM: unripe pulp mango, GPEM, unripe peel mango, RPUPA: ripe pulp papaya; RPEPA: ripe peel papaya, GPUPA: unripe pulp papaya, GPEPA, unripe peel papaya.

**Table 1:** Descriptive statistics including the mean and standard deviation for the selected parameters for pineapple, mango, and papaya (ripe and green) and for peel and pulp.

Type	Fe	Mn	Zn	Cu	Ca	Na	Mg	K	Pb
RPUP	0.98 ± 0.01	0.25 ± 0.02	0.47 ± 0.02	0.08 ± 0.01	10.37 ± 0.03	273.37 ± 1.41	14.82 ± 0.08	24.89 ± 0.51	0.95 ± 0.03
RPEP	0.81 ± 0.09	0.23 ± 0.01	0.43 ± 0.01	0.09 ± 0.01	11.77 ± 0.59	267.68 ± 7.17	24.72 ± 0.02	23.98 ± 0.43	0.84 ± 0.04
GPUP	0.78 ± 0.03	0.20 ± 0.02	0.39 ± 0.02	0.09 ± 0.01	10.36 ± 0.13	270.43 ± 7.82	25.10 ± 0.32	21.38 ± 0.70	0.82 ± 0.02
GPEP	0.88 ± 0.02	0.23 ± 0.01	0.43 ± 0.02	0.09 ± 0.01	11.77 ± 0.26	267.68 ± 3.37	24.72 ± 0.29	23.98 ± 0.38	0.84 ± 0.02
RPUM	0.60 ± 0.02	0.16 ± 0.02	0.14 ± 0.01	0.13 ± 0.02	4.36 ± 0.17	239.91 ± 9.40	14.58 ± 0.11	10.57 ± 0.99	0.26 ± 0.03
RPEM	0.40 ± 0.02	0.80 ± 0.02	0.12 ± 0.02	0.10 ± 0.01	6.09 ± 0.36	227.32 ± 7.35	14.69 ± 0.20	11.33 ± 0.42	0.08 ± 0.01
GPUM	0.38 ± 0.02	0.17 ± 0.02	0.22 ± 0.03	0.10 ± 0.01	5.12 ± 0.15	236.62 ± 10.07	13.43 ± 0.39	11.73 ± 1.74	0.07 ± 0.10
GPEM	0.60 ± 0.00	0.17 ± 0.02	0.21 ± 0.02	0.12 ± 0.01	8.16 ± 1.36	224.85 ± 27.29	15.31 ± 0.48	10.61 ± 0.72	0.23 ± 0.02
RPUPA	0.60 ± 0.00	0.01 ± 0.0	0.34 ± 0.06	0.07 ± 0.01	6.49 ± 0.10	429.79 ± 9.07	4.77 ± 0.08	4.19 ± 0.10	0.76 ± 0.13
RPEPA	0.60 ± 0.0	0.09 ± 0.07	2.75 ± 0.49	0.14 ± 0.03	18.94 ± 0.98	557.77 ± 6.84	4.97 ± 0.11	4.29 ± 0.29	0.81 ± 0.13
GPUPA	0.60 ± 0.0	0.23 ± 0.0	0.63 ± 0.01	0.10 ± 0.01	30.85 ± 0.71	632.06 ± 15.60	25.70 ± 0.42	4.54 ± 0.16	0.07 ± 0.03
GPEPA	0.60 ± 0.0	0.25 ± 0.02	0.90 ± 0.28	0.16 ± 0.01	30.10 ± 0.54	461.78 ± 45.32	25.35 ± 0.54	4.66 ± 0.0	0.19 ± 0.16

RPUP: ripe pulp pineapple; RPEP: ripe peel pineapple; GPUP: unripe pulp pineapple; GPEP: unripe peel pineapple; RPUM: ripe pulp mango; RPEM: ripe peel mango; GPUM: unripe pulp mango; GPEM: unripe peel mango; RPUPA: ripe pulp papaya; RPEPA: ripe peel papaya; GPUPA: unripe pulp papaya; GPEPA: unripe peel papaya.

**Table 2:** Proportion of variance explained by each discriminant function.

Function	LD1	LD2	LD3	LD4	LD5	LD6	LD7	LD8	LD9
Proportion of variance	0.5528	0.3205	0.0813	0.0298	0.0061	0.0055	0.0032	0.0007	0.0001

## 2 Experimental design, materials and methods

### 2.1 Sample preparation

Samples (twelve samples for each type and each stage of ripeness) of ripe and unripe fruits were purchased from a local market in Malaysia in March 2019. The samples were washed and peeled thoroughly before being cut into slices approximately 2 mm in diameter. The sliced fruit was then dried in a convection oven overnight at 60  °C. Next, a heavy duty grinder was used to grind the dried samples, followed by sieving to obtain refined flour. Airtight plastic was used to keep the flour at ambient temperature.

Next, 2 g peel and pulp samples were weighed into a crucible with the aid of preashing, and subsequently, a few drops of concentrated nitric acid were added. Next, the sample was placed into a muffle furnace for 6 h at 550  °C, and the furnace was allowed to cool to 200  °C before being opened. After that, a tong was used to place the crucible into a desiccator, and the crucible was allowed to cool to room temperature. The ash was then rinsed with nitric acid (HNO_3_) and filtered through filter paper into a 50 ml volumetric flask, and the final volume was made up with deionized water.

The third step involved the preparation of stock solutions of calcium, magnesium, potassium, iron, zinc, copper and lead. One milliliter of prepared stock solution was pipetted into a 100 ml volumetric flask and diluted to the mark with 0.1 M HNO_3_. The standard solution was used to prepare serial dilutions of 1 ppm, 2 ppm, 5 ppm, 8 ppm and 10 ppm in a 100 ml volumetric flask for calibration [2].

### 2.2 Sample analysis

Selected parameters (Ca, Mg, Na, K, Zn, Fe, Cu, and Pb) were determined in an air-acetylene flame by using an atomic absorption spectroscopy (AAS) instrument equipped with a hollow-cathode lamp (Perkin Elmer AAnalyst 100), and Mn was determined by using the periodate oxidation method (DR900).

### 2.4 Statistical analysis

**Fig. 1:**
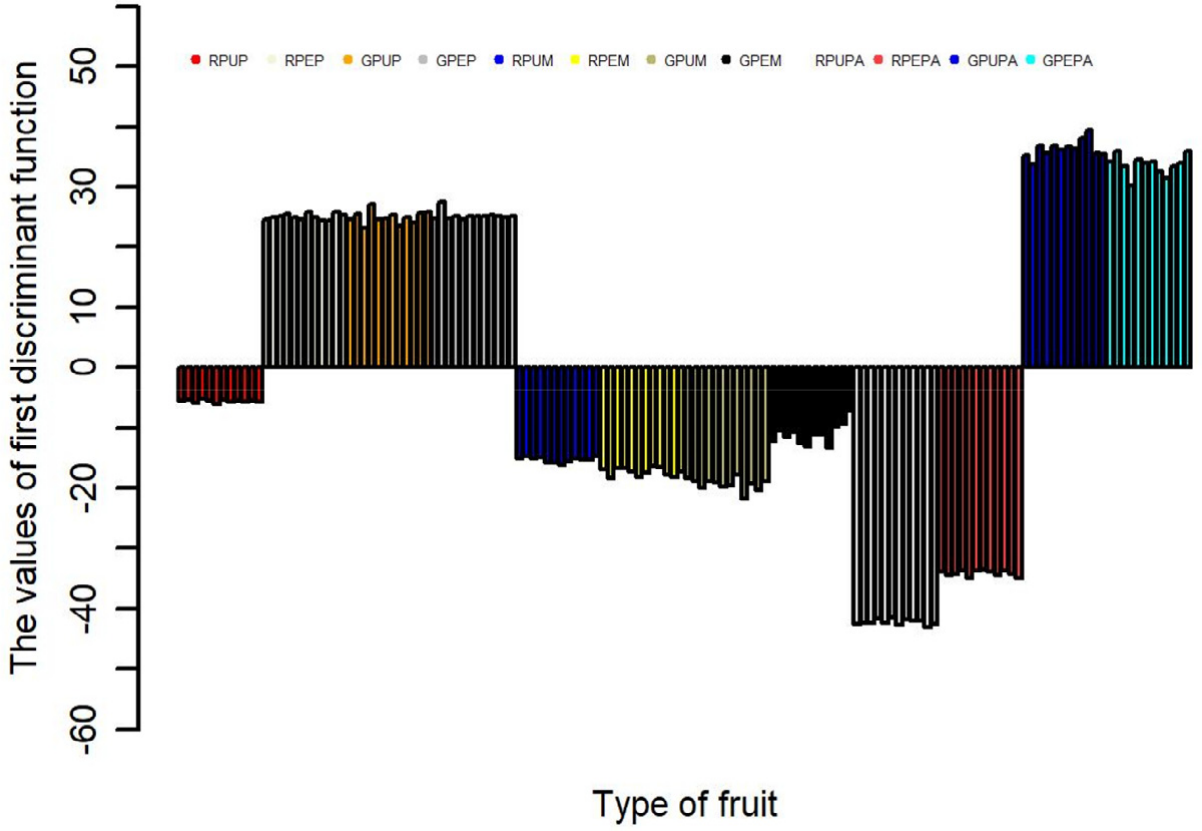
Showing the contribution of the first discriminant function on different samples.

**Fig. 2:**
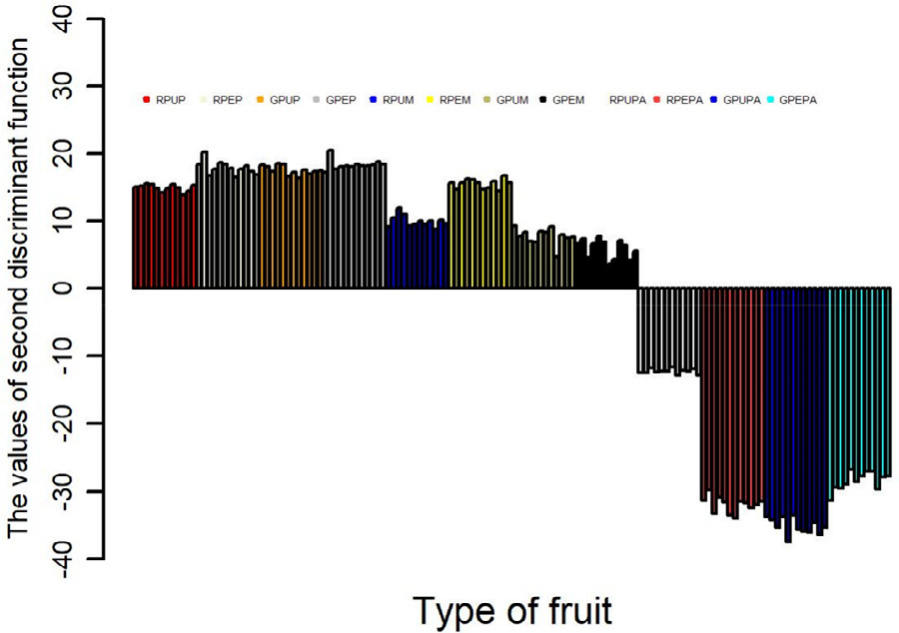
Showing the contribution of the second discriminant function on different samples.

**Fig. 3:**
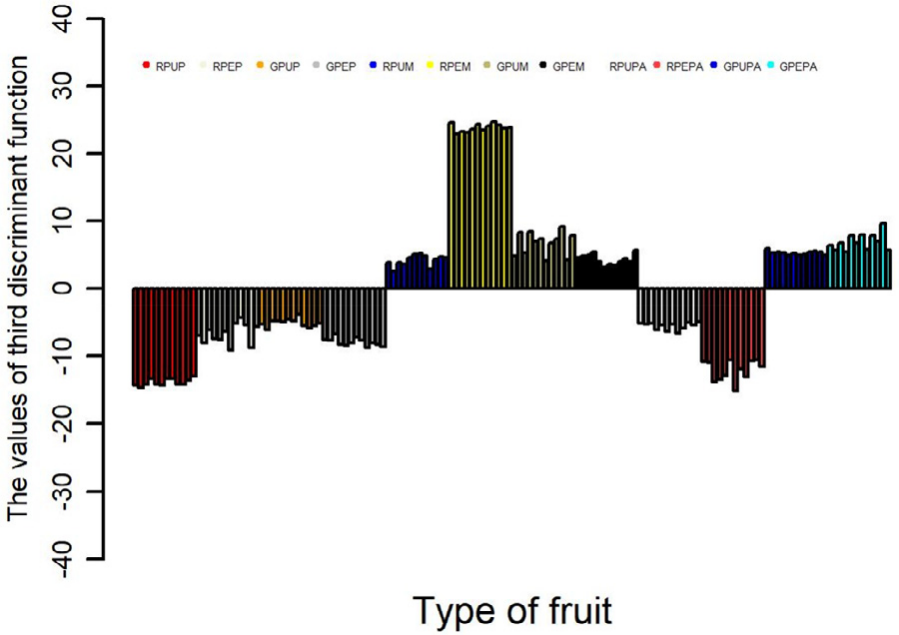
Showing the contribution of the third discriminant function on different samples.

**Fig. 4:**
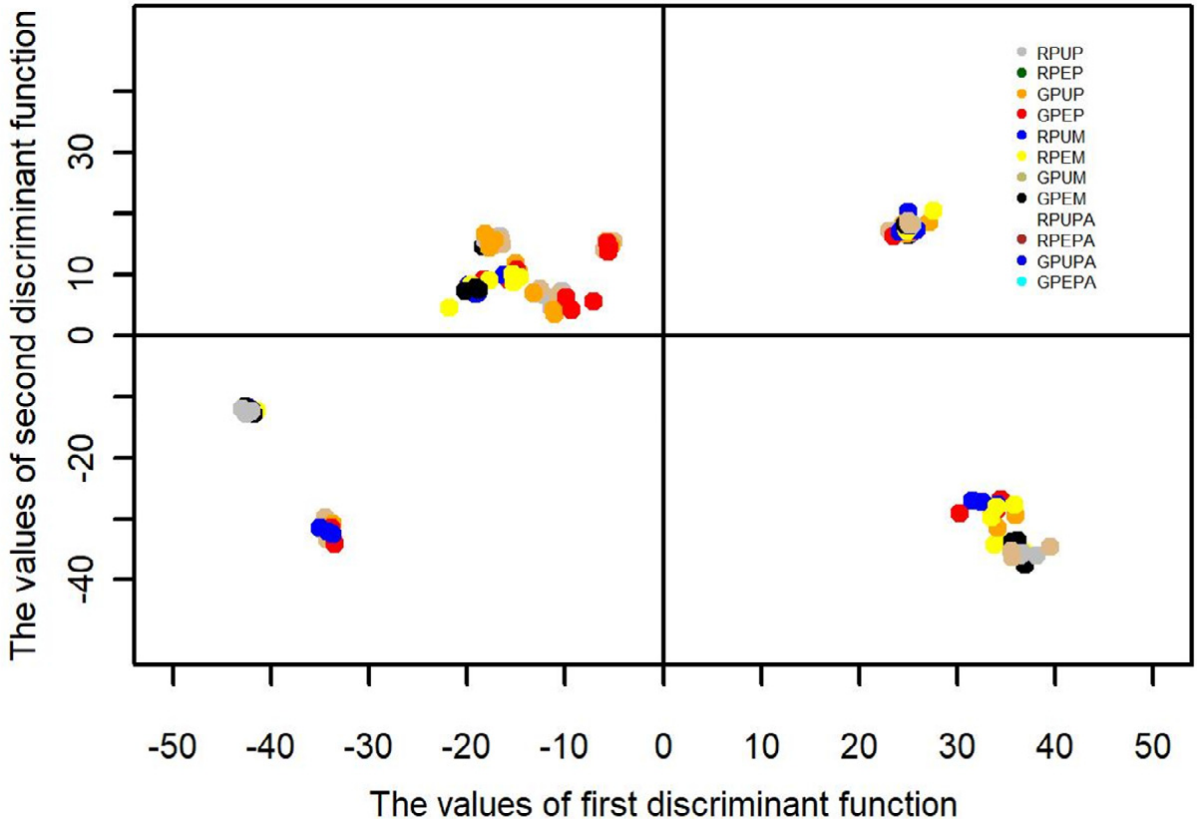
Showing the contribution of the first two discriminant functions on different samples.

**Fig. 5:**
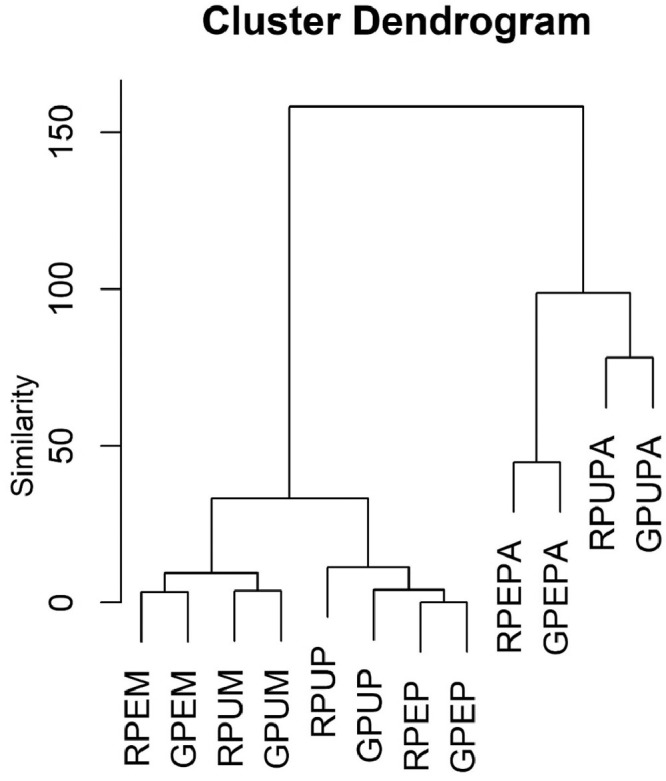
A dendrogram for mineral composition and pb in mango, papaya and pineapple.

The data obtained from three types of fruits and two stages of ripeness (ripe and unripe) for the major and minor mineral elements and one heavy metal were further analyzed using discriminant analysis [1,3] and cluster analysis [4,6]. The results of the discriminant function are presented in Table 2, showing the contribution of each discriminant function in separating different fruits and stages of ripeness. Furthermore, the contribution of each discriminant function for each sample is presented in Fig. 1, Fig. 2, Fig. 3, Fig. 4; the values of the discriminant functions show the differences among the three types of fruits and two stages of ripeness, which are mainly due to Mn and Fe. The results of cluster analysis for the different types of fruits, including two parts and stages of ripeness, are summarized in pictorial form using a distance matrix in Fig. 5, showing three different clusters.

## Appendix Supplementary materials

Image, application 1
