# Supplementary material for: Differentiation of ripe and unripe fruit flour using mineral composition data—Statistical assessment
Source: Data Brief. 2020 Mar 12;30:105414. doi: 10.1016/j.dib.2020.105414 (PMC7110322; doi:10.1016/j.dib.2020.105414)
Supplement: Supplementary file 1 [file mmc1.docx]

| Type | Fe | Mn | Zn | Cu | Ca | Na | Mg | K | Pb |
| --- | --- | --- | --- | --- | --- | --- | --- | --- | --- |
| RPUP | 0.99 | 0.24 | 0.49 | 0.08 | 10.38 | 273.7 | 14.81 | 25.09 | 0.92 |
| RPUP | 0.97 | 0.23 | 0.44 | 0.07 | 10.31 | 273.1 | 14.86 | 25.46 | 0.96 |
| RPUP | 0.95 | 0.26 | 0.48 | 0.08 | 10.38 | 275 | 14.67 | 25.95 | 0.99 |
| RPUP | 0.99 | 0.28 | 0.48 | 0.08 | 10.38 | 273.3 | 14.97 | 24.79 | 0.99 |
| RPUP | 0.99 | 0.24 | 0.45 | 0.07 | 10.4 | 273.3 | 14.82 | 24.87 | 0.92 |
| RPUP | 0.99 | 0.22 | 0.44 | 0.07 | 10.38 | 272.5 | 14.7 | 24.39 | 0.9 |
| RPUP | 0.95 | 0.25 | 0.48 | 0.09 | 10.37 | 274 | 14.93 | 24.9 | 0.95 |
| RPUP | 0.98 | 0.27 | 0.48 | 0.08 | 10.33 | 270.6 | 14.83 | 25.03 | 0.93 |
| RPUP | 0.99 | 0.24 | 0.45 | 0.08 | 10.38 | 273.5 | 14.82 | 24.99 | 0.93 |
| RPUP | 0.97 | 0.21 | 0.44 | 0.07 | 10.35 | 272.2 | 14.81 | 24.01 | 0.96 |
| RPUP | 0.98 | 0.25 | 0.49 | 0.09 | 10.4 | 276.3 | 14.83 | 24.29 | 0.98 |
| RPUP | 0.98 | 0.28 | 0.47 | 0.08 | 10.38 | 272.9 | 14.82 | 24.86 | 0.94 |
| RPEP | 0.83 | 0.23 | 0.43 | 0.09 | 11.12 | 281 | 24.72 | 23.98 | 0.78 |
| RPEP | 0.88 | 0.24 | 0.42 | 0.1 | 11.05 | 264.8 | 24.73 | 24.95 | 0.83 |
| RPEP | 0.77 | 0.22 | 0.43 | 0.08 | 12.35 | 260.2 | 24.74 | 23.61 | 0.8 |
| RPEP | 0.83 | 0.24 | 0.44 | 0.09 | 12.24 | 272.6 | 24.72 | 24.48 | 0.85 |
| RPEP | 0.84 | 0.23 | 0.43 | 0.09 | 11.31 | 270.3 | 24.72 | 24.01 | 0.89 |
| RPEP | 0.83 | 0.24 | 0.43 | 0.1 | 11.18 | 275 | 24.72 | 23.59 | 0.8 |
| RPEP | 0.96 | 0.22 | 0.43 | 0.07 | 12.32 | 261.3 | 24.73 | 23.36 | 0.88 |
| RPEP | 0.7 | 0.24 | 0.45 | 0.09 | 12.12 | 272.5 | 24.71 | 23.8 | 0.86 |
| RPEP | 0.64 | 0.23 | 0.45 | 0.09 | 11.26 | 263.4 | 24.75 | 24.09 | 0.83 |
| RPEP | 0.72 | 0.23 | 0.42 | 0.1 | 11.42 | 258.1 | 24.7 | 24.23 | 0.81 |
| RPEP | 0.91 | 0.21 | 0.43 | 0.07 | 12.65 | 260.9 | 24.74 | 23.99 | 0.8 |
| RPEP | 0.75 | 0.25 | 0.44 | 0.11 | 12.17 | 272 | 24.7 | 23.65 | 0.92 |
| GPUP | 0.77 | 0.2 | 0.4 | 0.09 | 10.36 | 256 | 24.97 | 21.89 | 0.85 |
| GPUP | 0.78 | 0.18 | 0.41 | 0.1 | 10.35 | 270.6 | 25.22 | 22.41 | 0.81 |
| GPUP | 0.75 | 0.2 | 0.37 | 0.08 | 10.13 | 273.1 | 24.59 | 21.47 | 0.8 |
| GPUP | 0.76 | 0.22 | 0.37 | 0.09 | 10.56 | 274.9 | 25.75 | 22.57 | 0.82 |
| GPUP | 0.77 | 0.21 | 0.4 | 0.1 | 10.21 | 261.2 | 25.01 | 21.92 | 0.83 |
| GPUP | 0.75 | 0.18 | 0.41 | 0.09 | 10.43 | 266.2 | 25.1 | 20.71 | 0.81 |
| GPUP | 0.77 | 0.2 | 0.38 | 0.08 | 10.36 | 279.2 | 25.27 | 21.24 | 0.8 |
| GPUP | 0.76 | 0.22 | 0.38 | 0.09 | 10.35 | 279.9 | 24.72 | 20.35 | 0.84 |
| GPUP | 0.79 | 0.18 | 0.41 | 0.1 | 10.41 | 260.6 | 25.12 | 21.36 | 0.82 |
| GPUP | 0.8 | 0.18 | 0.41 | 0.09 | 10.36 | 272.4 | 24.8 | 21.18 | 0.83 |
| GPUP | 0.82 | 0.19 | 0.37 | 0.08 | 10.18 | 278.3 | 25.32 | 20.66 | 0.84 |
| GPUP | 0.83 | 0.21 | 0.38 | 0.08 | 10.57 | 272.7 | 25.34 | 20.82 | 0.84 |
| GPEP | 0.86 | 0.21 | 0.41 | 0.09 | 11.82 | 270.5 | 24.62 | 23.23 | 0.83 |
| GPEP | 0.9 | 0.23 | 0.43 | 0.09 | 10.95 | 264.4 | 25.63 | 24.12 | 0.82 |
| GPEP | 0.87 | 0.25 | 0.42 | 0.09 | 11.81 | 270.5 | 24.64 | 23.15 | 0.83 |
| GPEP | 0.9 | 0.23 | 0.44 | 0.11 | 11.86 | 276 | 24.61 | 24.09 | 0.84 |
| GPEP | 0.88 | 0.22 | 0.4 | 0.08 | 11.87 | 265.5 | 24.5 | 24.29 | 0.83 |
| GPEP | 0.86 | 0.22 | 0.44 | 0.09 | 11.82 | 266.5 | 24.67 | 24.08 | 0.86 |
| GPEP | 0.85 | 0.25 | 0.43 | 0.09 | 11.82 | 269.5 | 24.68 | 24.13 | 0.88 |
| GPEP | 0.87 | 0.23 | 0.44 | 0.11 | 11.85 | 266.4 | 24.68 | 24.03 | 0.84 |
| GPEP | 0.89 | 0.21 | 0.4 | 0.09 | 11.88 | 264.5 | 24.68 | 24.18 | 0.87 |
| GPEP | 0.88 | 0.23 | 0.43 | 0.09 | 11.93 | 265.4 | 24.63 | 24.16 | 0.85 |
| GPEP | 0.92 | 0.24 | 0.43 | 0.09 | 11.8 | 266.5 | 24.62 | 24.19 | 0.82 |
| GPEP | 0.91 | 0.22 | 0.44 | 0.09 | 11.86 | 266.5 | 24.65 | 24.14 | 0.83 |
| RPUM | 0.62 | 0.122 | 0.1452 | 0.1586 | 4.32 | 233.6 | 14.667 | 9.55 | 0.2562 |
| RPUM | 0.64 | 0.144 | 0.1398 | 0.1209 | 4.37 | 243.6 | 14.655 | 11.33 | 0.2713 |
| RPUM | 0.58 | 0.188 | 0.1395 | 0.1291 | 3.915 | 245 | 14.657 | 12.29 | 0.287 |
| RPUM | 0.57 | 0.158 | 0.1189 | 0.1129 | 4.37 | 242.4 | 14.667 | 12.27 | 0.1827 |
| RPUM | 0.58 | 0.162 | 0.1482 | 0.1538 | 4.426 | 241.4 | 14.405 | 10.1 | 0.3038 |
| RPUM | 0.57 | 0.174 | 0.131 | 0.1197 | 4.204 | 246.4 | 14.469 | 9.99 | 0.2876 |
| RPUM | 0.6 | 0.168 | 0.1378 | 0.118 | 4.462 | 214.3 | 14.412 | 9.55 | 0.2617 |
| RPUM | 0.6 | 0.184 | 0.14915 | 0.1164 | 4.421 | 249.7 | 14.527 | 10.13 | 0.24 |
| RPUM | 0.6 | 0.122 | 0.1453 | 0.1542 | 4.442 | 232.8 | 14.534 | 11.23 | 0.2617 |
| RPUM | 0.6 | 0.144 | 0.137 | 0.1126 | 4.469 | 243.9 | 14.648 | 9.58 | 0.2177 |
| RPUM | 0.6 | 0.188 | 0.1448 | 0.1276 | 4.419 | 244.7 | 14.586 | 10.66 | 0.2508 |
| RPUM | 0.6 | 0.168 | 0.13971 | 0.143928 | 4.55 | 241.1 | 14.763 | 10.17 | 0.2876 |
| RPEM | 0.4 | 0.832 | 0.1146 | 0.11 | 6.15 | 235.2 | 14.832 | 11.34 | 0.07111 |
| RPEM | 0.38 | 0.775 | 0.1018 | 0.1017 | 6.12 | 236.14 | 14.274 | 11.6 | 0.08647 |
| RPEM | 0.42 | 0.786 | 0.1022 | 0.1026 | 6.13 | 224.3 | 14.866 | 11.31 | 0.07599 |
| RPEM | 0.42 | 0.8 | 0.1226 | 0.1194 | 6.14 | 225.23 | 14.802 | 12.12 | 0.07592 |
| RPEM | 0.42 | 0.82 | 0.1261 | 0.0818 | 6.27 | 222.4 | 14.671 | 11.68 | 0.0802 |
| RPEM | 0.43 | 0.825 | 0.1445 | 0.1159 | 6.12 | 218.3 | 14.461 | 10.68 | 0.08701 |
| RPEM | 0.39 | 0.786 | 0.1477 | 0.1064 | 6.6 | 219.13 | 14.587 | 11.27 | 0.07749 |
| RPEM | 0.38 | 0.8 | 0.1289 | 0.1057 | 6.08 | 243.08 | 14.952 | 11.17 | 0.07697 |
| RPEM | 0.41 | 0.832 | 0.1036 | 0.1004 | 6.19 | 228 | 14.957 | 11.03 | 0.0837 |
| RPEM | 0.37 | 0.785 | 0.1311 | 0.119 | 6.16 | 227 | 14.569 | 10.69 | 0.07677 |
| RPEM | 0.38 | 0.786 | 0.1246 | 0.09714 | 5.03 | 226 | 14.624 | 11.32 | 0.07617 |
| RPEM | 0.37 | 0.7933 | 0.122 | 0.0961 | 6.13 | 223.05 | 14.729 | 11.7 | 0.07106 |
| GPUM | 0.36 | 0.152 | 0.2351 | 0.101 | 5.103 | 242.1 | 13.61 | 14.54 | 0.03965 |
| GPUM | 0.36 | 0.198 | 0.2402 | 0.0954 | 4.836 | 249.5 | 13.77 | 11.16 | 0.02566 |
| GPUM | 0.38 | 0.148 | 0.1694 | 0.1034 | 5.252 | 234.3 | 13.15 | 13.42 | 0.02194 |
| GPUM | 0.38 | 0.185 | 0.2202 | 0.0993 | 5.133 | 234.7 | 13.71 | 10.17 | 0.04486 |
| GPUM | 0.38 | 0.152 | 0.2279 | 0.1038 | 4.957 | 245.3 | 13.58 | 10.84 | 0.04773 |
| GPUM | 0.42 | 0.198 | 0.2444 | 0.0982 | 5.003 | 230.9 | 13.41 | 11.33 | 0.02871 |
| GPUM | 0.38 | 0.148 | 0.1752 | 0.1026 | 5.297 | 232 | 13.03 | 13.51 | 0.2625 |
| GPUM | 0.38 | 0.185 | 0.2255 | 0.1087 | 5.028 | 237.9 | 13.94 | 12.92 | 0.02763 |
| GPUM | 0.44 | 0.152 | 0.2319 | 0.0845 | 5.132 | 247.4 | 12.8 | 8.37 | 0.01148 |
| GPUM | 0.37 | 0.198 | 0.2374 | 0.1076 | 5.178 | 211 | 13.71 | 10.34 | 0.03734 |
| GPUM | 0.38 | 0.148 | 0.1685 | 0.0854 | 5.379 | 235.5 | 12.8 | 12.73 | 0.3056 |
| GPUM | 0.37 | 0.185 | 0.2215 | 0.1052 | 5.167 | 238.8 | 13.67 | 11.45 | 0.01974 |
| GPEM | 0.6 | 0.153 | 0.2175 | 0.1331 | 6.58 | 241.5 | 15.28 | 9.75 | 0.19349 |
| GPEM | 0.6 | 0.174 | 0.2204 | 0.1313 | 7.43 | 215.5 | 15.7 | 10.39 | 0.2446 |
| GPEM | 0.6 | 0.166 | 0.1644 | 0.1063 | 8.96 | 203.34 | 15.14 | 9.59 | 0.2263 |
| GPEM | 0.6 | 0.197 | 0.2279 | 0.12 | 9.52 | 159.9 | 15.31 | 10.84 | 0.2301 |
| GPEM | 0.6 | 0.153 | 0.2079 | 0.1338 | 6.37 | 233.66 | 15.12 | 10.63 | 0.2118 |
| GPEM | 0.6 | 0.174 | 0.2004 | 0.1309 | 7.29 | 245.5 | 14.64 | 11.84 | 0.2492 |
| GPEM | 0.6 | 0.166 | 0.1969 | 0.1046 | 9.17 | 242.22 | 15.05 | 10.48 | 0.2334 |
| GPEM | 0.6 | 0.197 | 0.2332 | 0.1193 | 9.76 | 239.2 | 14.84 | 12.08 | 0.2297 |
| GPEM | 0.6 | 0.153 | 0.2109 | 0.1282 | 6.46 | 239.3 | 14.9 | 10.36 | 0.2209 |
| GPEM | 0.6 | 0.174 | 0.2135 | 0.1278 | 7.43 | 246.3 | 15.77 | 10.34 | 0.2479 |
| GPEM | 0.6 | 0.166 | 0.1618 | 0.1026 | 9.1 | 241.4 | 15.62 | 10.59 | 0.2325 |
| GPEM | 0.6 | 0.197 | 0.2188 | 0.1254 | 9.86 | 190.32 | 16.34 | 10.37 | 0.2496 |
| RPUPA | 0.6 | 0.001 | 0.4332 | 0.07731 | 6.476 | 434.6 | 4.782 | 4.146 | 0.6261 |
| RPUPA | 0.6 | 0.001 | 0.2876 | 0.05004 | 6.498 | 433.5 | 4.772 | 4.052 | 0.6942 |
| RPUPA | 0.6 | 0.0013 | 0.3388 | 0.05995 | 6.361 | 422.4 | 4.845 | 4.303 | 0.6677 |
| RPUPA | 0.6 | 0.0012 | 0.3302 | 0.07877 | 6.443 | 436.5 | 4.832 | 4.165 | 0.893 |
| RPUPA | 0.6 | 0.0013 | 0.4261 | 0.0847 | 6.503 | 430.5 | 4.794 | 4.298 | 0.6906 |
| RPUPA | 0.6 | 0.001 | 0.2772 | 0.05781 | 6.502 | 429 | 4.813 | 4.059 | 0.9725 |
| RPUPA | 0.6 | 0.0012 | 0.3376 | 0.06228 | 6.301 | 420.7 | 4.731 | 4.282 | 0.6954 |
| RPUPA | 0.6 | 0.001 | 0.3234 | 0.07883 | 6.554 | 445.4 | 4.66 | 4.222 | 0.9527 |
| RPUPA | 0.6 | 0.0012 | 0.4214 | 0.08386 | 6.633 | 419.7 | 4.785 | 4.129 | 0.849 |
| RPUPA | 0.6 | 0.001 | 0.2768 | 0.05587 | 6.629 | 423.3 | 4.867 | 4.089 | 0.7057 |
| RPUPA | 0.6 | 0.0012 | 0.3145 | 0.06941 | 6.466 | 418.8 | 4.566 | 4.35 | 0.7195 |
| RPUPA | 0.6 | 0.001 | 0.3252 | 0.07908 | 6.484 | 443.1 | 4.746 | 4.132 | 0.6063 |
| RPEPA | 0.6 | 0.121 | 2.272 | 0.09494 | 18.96 | 553.8 | 4.973 | 4.374 | 0.8792 |
| RPEPA | 0.6 | 0.18 | 2.891 | 0.1156 | 18.54 | 547.6 | 4.95 | 4.368 | 0.9701 |
| RPEPA | 0.6 | 0.048 | 2.994 | 0.153 | 19.48 | 557.1 | 4.821 | 4.365 | 0.784 |
| RPEPA | 0.6 | 0.013 | 2.076 | 0.1355 | 17.41 | 558.7 | 5.013 | 4.377 | 1.027 |
| RPEPA | 0.6 | 0.122 | 3.321 | 0.1069 | 18.88 | 563.6 | 4.868 | 4.376 | 0.7557 |
| RPEPA | 0.6 | 0.152 | 2.87 | 0.1404 | 20.21 | 558.9 | 4.993 | 3.373 | 0.8442 |
| RPEPA | 0.6 | 0.023 | 3.15 | 0.1983 | 19.73 | 559.8 | 4.871 | 4.364 | 0.9069 |
| RPEPA | 0.6 | 0.012 | 2.071 | 0.138 | 17.7 | 565 | 5.199 | 4.369 | 0.678 |
| RPEPA | 0.6 | 0.111 | 3.31 | 0.1075 | 19.34 | 545.6 | 4.965 | 4.376 | 0.7918 |
| RPEPA | 0.6 | 0.133 | 2.874 | 0.1499 | 19.71 | 559.3 | 5.121 | 4.374 | 0.6605 |
| RPEPA | 0.6 | 0.18 | 3.155 | 0.1904 | 19.88 | 554.2 | 4.909 | 4.368 | 0.7774 |
| RPEPA | 0.6 | 0.012 | 2.068 | 0.1339 | 17.44 | 569.6 | 4.937 | 4.369 | 0.5888 |
| GPUPA | 0.6 | 0.235 | 0.6166 | 0.09913 | 30.44 | 598.8 | 25.5 | 4.287 | 0.08756 |
| GPUPA | 0.6 | 0.23 | 0.6218 | 0.09458 | 29.85 | 629.6 | 25.1 | 4.458 | 0.05096 |
| GPUPA | 0.6 | 0.239 | 0.6217 | 0.1025 | 31.03 | 637 | 25.82 | 4.58 | 0.08218 |
| GPUPA | 0.6 | 0.233 | 0.6336 | 0.1075 | 29.88 | 630.7 | 25.63 | 4.65 | 0.09941 |
| GPUPA | 0.6 | 0.233 | 0.6209 | 0.1048 | 32.09 | 644.7 | 25.58 | 4.394 | 0.09241 |
| GPUPA | 0.6 | 0.23 | 0.6362 | 0.09114 | 30.34 | 611.3 | 25.73 | 4.86 | 0.10125 |
| GPUPA | 0.6 | 0.234 | 0.6291 | 0.1018 | 31.17 | 639 | 25.71 | 4.73 | 0.09456 |
| GPUPA | 0.6 | 0.234 | 0.6498 | 0.1086 | 30.76 | 654.6 | 25.64 | 4.32 | 0.09941 |
| GPUPA | 0.6 | 0.234 | 0.6214 | 0.102 | 31.55 | 637.8 | 26.13 | 4.554 | 0.05616 |
| GPUPA | 0.6 | 0.231 | 0.6362 | 0.08403 | 30.58 | 647.3 | 26.75 | 4.583 | 0.02997 |
| GPUPA | 0.6 | 0.236 | 0.6265 | 0.107 | 31.83 | 620.8 | 25.32 | 4.574 | 0.02781 |
| GPUPA | 0.6 | 0.22 | 0.6408 | 0.1063 | 30.66 | 633.1 | 25.44 | 4.544 | 0.07034 |
| GPEPA | 0.6 | 0.282 | 0.9473 | 0.1656 | 29.31 | 597.9 | 25.39 | 4.66 | 0.1215 |
| GPEPA | 0.6 | 0.194 | 0.9895 | 0.1568 | 30.5 | 466.9 | 25.99 | 4.662 | 0.1414 |
| GPEPA | 0.6 | 0.247 | 0.9924 | 0.1713 | 30.63 | 468.6 | 25.22 | 4.666 | 0.136 |
| GPEPA | 0.6 | 0.256 | 0.9456 | 0.1497 | 30.32 | 447.4 | 24.05 | 4.661 | 0.4447 |
| GPEPA | 0.6 | 0.254 | 0.9705 | 0.1728 | 29.51 | 446.3 | 25.86 | 4.661 | 0.1075 |
| GPEPA | 0.6 | 0.223 | 0.9905 | 0.1538 | 30.31 | 449.6 | 25.55 | 4.663 | 0.07518 |
| GPEPA | 0.6 | 0.244 | 1.01 | 0.1761 | 30.16 | 440.5 | 25.72 | 4.667 | 0.01543 |
| GPEPA | 0.6 | 0.244 | 0.9657 | 0.1436 | 30.14 | 410.8 | 24.91 | 4.653 | 0.4604 |
| GPEPA | 0.6 | 0.263 | 0.9667 | 0.1729 | 29.23 | 452.4 | 24.98 | 4.663 | 0.09725 |
| GPEPA | 0.6 | 0.254 | 0.991 | 0.1577 | 31.01 | 459.4 | 25.21 | 4.663 | 0.1317 |
| GPEPA | 0.6 | 0.243 | 0.011 | 0.174 | 29.82 | 453.2 | 25.48 | 4.667 | 0.1118 |
| GPEPA | 0.6 | 0.245 | 0.9666 | 0.1415 | 30.21 | 448.3 | 25.87 | 4.657 | 0.4604 |

RPUP: ripe pulp pineapple; RPEP: ripe peel pineapple, GPUP: unripe pulp pineapple, GPEP, unripe peel pineapple, RPUM: ripe pulp mango; RPEM: ripe peel mango, GPUM: unripe pulp mango, GPEM, unripe peel mango, RPUPA: ripe pulp papaya; RPEPA: ripe peel papaya, GPUPA: unripe pulp papaya, GPEPA, unripe peel papaya.
